# First Report of ‘*Candidatus* Mycoplasma haematomacacae’ in Laboratory-Kept Rhesus Monkeys (*Macaca mulatta*) Maintained in Rio de Janeiro, Brazil

**DOI:** 10.3390/vetsci9080443

**Published:** 2022-08-19

**Authors:** Anna Claudia Baumel Mongruel, André Tavares Somma, Ana Cristina Araújo Pinto, Carla de Freitas Campos, Mônica Ingeborg Zuege Calado, Fabiano Montiani-Ferreira, Thállitha Samih Wischral Jayme Vieira, Rafael Felipe da Costa Vieira

**Affiliations:** 1 Vector-Borne Diseases Laboratory, Department of Veterinary Medicine, Universidade Federal do Paraná, Curitiba 80035-050, Brazil; 2 Department of Veterinary Medicine, Universidade Federal do Paraná, Curitiba 80035-050, Brazil; 3 Instituto de Ciência e Tecnologia em Biomodelos, Fundação Oswaldo Cruz, Rio de Janeiro 21040-900, Brazil; 4 Leonard M. Miller School of Medicine, University of Miami, Miami, FL 33136, USA; 5 Global One Health initiative (GOHi), The Ohio State University, Columbus, OH 43210, USA

**Keywords:** laboratory animals, hemotropic mycoplasmas, hemotropic *Mycoplasma* spp., genetic diversity, animal experimentation

## Abstract

**Simple Summary:**

Health assessment in animals used in research studies are essential, since only animals that present no diseases are considered suitable for these purposes. In laboratory kept animals, a bacterium that infects red blood cells, named hemotropic *Mycoplasma* (also called hemoplasmas), has been described as problem for research studies. Different hemoplasma species have been detected infecting monkeys from Brazil. However, the occurrence of these bacteria in monkeys maintained in laboratory in Brazil have never been described. Accordingly, this study aimed: (1) to screen laboratory-kept rhesus monkeys for hemoplasmas; (2) to verify if any of the hemoplasma-positive animals demonstrate a decrease in their red blood cells counts; and (3) to investigate the genetic diversity of hemoplasma species in monkeys from Brazil. Five out of eight (62.5%) rhesus monkeys tested positive for hemoplasmas using a technique that detects DNA from these bacteria in monkey’s blood. Further analysis demonstrated that rhesus monkeys were infected by a species named ‘*Candidatus* Mycoplasma haematomacacae’ that had already been described occurring in monkeys from Japan and USA. Although no decreases on red blood cells count were perceived in rhesus monkeys evaluated herein, future studies are needed to elucidate if ‘*Ca*. M. haematomacacae’ is a problem for research studies that use rhesus monkeys.

**Abstract:**

Health monitoring programs in animals used as experimental models are essential, since only disease-free subjects are considered suitable for research purposes. In laboratory-kept animals, hemoplasmas have been described as an important confounding variable. Different hemoplasma species have been detected infecting non-human primates (NHP) from Brazil. However, the occurrence of hemoplasma species in laboratory-kept NHP in Brazil has not-yet been assessed. Accordingly, this study aimed (i) to screen laboratory-kept rhesus monkeys for hemoplasmas, (ii) to verify if any of the hemoplasma-positive animals demonstrate hematological abnormalities, and (iii) to assess the genotype diversity of hemoplasma species in NHP from Brazil. Five out of eight (62.5%; 95% CI: 3.05–8.63) rhesus monkeys tested positive for hemotropic *Mycoplasma* spp. by PCR. Sequencing, phylogenetic, distance, and genotype diversity analyses of partial 16S rRNA gene demonstrate that rhesus monkeys were infected by ‘*Candidatus* Mycoplasma haematomacacae’ (formerly ‘*Candidatus* Mycoplasma haemomacaque’). Assessments of partial 16S rRNA diversity of hemoplasma species in NHP suggest that at least four genetically diverse groups may occur in Brazil. Although no hematological abnormalities were demonstrated in rhesus monkeys evaluated herein, future studies are needed to elucidate the influence of ‘*Ca*. M. haematomacacae’ as a confounding variable on research studies.

## 1. Introduction

The use of animal experimentation in biomedical research has vastly increased scientific knowledge of human health and disease [1]. Laboratory animal health monitoring and pathogen detection programs are essential, since only disease-free animals that may modify its physiological parameters are considered suitable for research purposes [2]. It is estimated that 100,000 to 200,000 non-human primates (NHP) are used in research worldwide annually, mainly on microbiology, neurosciences, and biochemistry/chemistry investigations [3]. Rhesus monkeys (*Macaca mulatta*) are the most common NHP used in biomedical research [4]. Recently, this NHP species has been used especially as a model for COVID-19 [5] and scrub typhus transmission studies [6].

Different pathogens affect laboratory-kept animals. The Mollicutes class comprises approximately 200 bacteria species, including mucosal and hemotropic mycoplasmas (hemoplasmas) [7,8]. Mucosal infections by *Mycoplasma* spp. have been well documented through the years in laboratories [9,10,11,12,13]. In fact, the occurrence of *Mycoplasma pulmonis* in laboratory rats is associated with higher levels of inflammation parameters and immune system activation, which may represent a potential bias for research interpretations [14].

In relation to the hemotropic *Mycoplasma* species, these are epicellular bacteria that are able to attach to the surface of mammal’s erythrocytes and may cause infectious anemia [15]. In laboratory rodents, *Mycoplasma coccoides* (formerly *Eperythrozoon coccoides*) and *Mycoplasma haemomuris* (formerly *Haemobartonella muris*) were described as a type of important confounding variable [16,17] in studies on *Plasmodium chabaudi* [18], *Trypanosoma brucei* [19], *Plasmodium berghei* [20,21], viral infections [22], and cancer studies [23]. Additionally, the suitability of sheep and beagle dogs for research on biomedical purposes has also raised concerns due to subclinical and acute hemoplasma infections, especially on splenectomised animals [24,25]. 

Regarding the occurrence of hemoplasmas in NHP, three species are described: ‘*Candidatus* Mycoplasma kahanei’ [26], ‘*Candidatus* Mycoplasma aoti’ [27], and ‘*Candidatus* Mycoplasma haematomacacae’ (formerly ‘*Candidatus* Mycoplasma haemomacaque’) [28,29]. Additionally, potentially novel hemotropic *Mycoplasma* sp. closely related to ‘*Ca.* M. kahanei’ [30,31], and to ‘*Ca.* M. haematomacacae’ [32,33,34] have been described in NHP from Brazil. However, hemoplasma infection in NHP used for research purposes have not been assessed to date. Accordingly, this study aimed (i) to screen laboratory-kept rhesus monkeys for hemotropic *Mycoplasma* spp., (ii) to verify if any of the positive animals sampled herein demonstrate hematological abnormalities, and (iii) to assesses the genotype diversity of hemoplasma species in NHP from Brazil based on partial 16S rRNA gene and compare with sequences from the present study. 

## 2. Material and Methods

### 2.1. Ethical Approval

This study was approved by the Ethics Committee for Animal Experimentation and Animal Welfare at the Fundação Oswaldo Cruz (Fiocruz) (Primatology’s License number LW-5/16) and conducted according to the ethical principles of animal experimentation, adopted by the Brazilian College of Animal Experimentation.

### 2.2. Sampling

A rhesus monkey (*Macaca mulatta*) laboratory colony was evaluated in two different moments. EDTA (ethylenediamine-tetraacetic acid) blood samples were obtained at two time points (T): T1—February 2018; and T2—December 2018. The laboratory colony is maintained at a certified Research Center in Rio de Janeiro City, southeastern Brazil (22.8830° S, 43.2458° W), for use on studies focusing on arboviruses.

In T1, a total of eight rhesus monkeys were evaluated. In T2, only six animals were re-sampled since two of them died. Rhesus monkeys were inspected for ectoparasites (ticks, fleas, and lice) by close visual inspection, and blood samples collected (up to 5 mL) by saphenous venipuncture.

### 2.3. Packed Cell Volume and White Blood Cell Count

The packed cell volume (PCV) and white blood cell (WBC) count were assessed for animals in T2, and samples stored at −20 °C until molecular analysis. A PCV of <0.37 L/L was used as indicator of anemia for males while a PCV of <0.36 L/L was used for females [35]. For WBC count, values >15,660 cells/mm^3^ were considered as an indicator of leukocytosis [36].

### 2.4. DNA Extraction and Polymerase Chain Reaction (PCR) Assays

DNA was extracted from all EDTA blood samples using a commercial kit (llustra^TM^ blood GenomicPrep Mini Spin Kit, GE Healthcare, Little Chalfont, UK), according to the manufacturer’s instructions. A conventional PCR for the mammal-endogenous gene glyceraldehyde-3-phosphate dehydrogenase (*gapdh*) was performed in all samples to monitor DNA extraction [37]. Thereafter, DNA samples were screened for hemoplasmas using a previously described PCR protocol targeting the 16S rRNA gene (∼900 bp) [38,39]. Rhesus monkey DNA samples that tested positive in the PCR assay based on the 16S rRNA gene were subjected to PCR assay targeting a fragment (800 pb) of the 23S rRNA gene from hemoplasmas [40]. ‘*Candidatus* Mycoplasma haematominutum’ (formerly ‘*Candidatus* Mycoplasma haemominutum’) DNA obtained from a naturally infected cat (*Felis catus*) [41] and ultrapure water were used as positive and negative controls, respectively.

### 2.5. Sequencing, Phylogenetic, Genotype Diversity, and Distance Analysis

Amplicons obtained from three hemotropic *Mycoplasma* sp.-positive samples were purified by enzymatic purification (ExoSAP-IT™ PCR Product Cleanup Reagent, Thermo Scientific, Waltham, MA, USA) and sequenced in both directions by Sanger method (3500 Genetic Analyzer, Applied Biosystems, Foster City, CA, USA), with nucleotide sequences of the 16S rRNA gene submitted to the GenBank^®^ database. Thereafter, sequences were subjected to BLASTn analysis [42] for determining the identity with sequences deposited in GenBank^®^ database. Sequences were then aligned using MAFFT [43], and improved on GUIDANCE2 [44]. Best-fit evolutionary model was estimated as TPM3uf+I+G by jModeltest 2.1.4 [45]. The Bayesian information criterion (BIC) algorithm was used for phylogenetic inference and made by MrBayes on XSEDE using Cipres Portal (https://www.phylo.org) (accessed on 2 June 2022), with two independent runs of 20,000,000 MCMC and 5% of burn-in. Reconstruction was visualized with FigTree 1.4.0 software.

Genotype diversity among 16S rRNA gene sequences detected herein and NHP closely related hemoplasmas were aligned, as described above, and submitted for analysis on DnaSP6 software [46]. Inference and graphic representation were made by TCS Network method [47] on PopART software [48], respectively. Furthermore, a distance analysis based on a split-network was made using Splitstree v. 4.14.6 software [49] applying Neighbor-Net method with the same alignment. 

## 3. Results

No rhesus monkeys evaluated herein presented ectoparasites (ticks, fleas, and lice) at the time of sampling. The mean PCV for all hemoplasma-positive rhesus monkeys was 0.43 L/L. None of the hemotropic *Mycoplasma* spp.-positive rhesus monkeys were considered anemic. Regarding WBC count, the mean value for all hemoplasma-positive rhesus monkeys was 12,675 cells/mm^3^.

The mammal-endogenous *gapdh* gene was successfully amplified in all DNA samples from T1 and T2. In T1, five out of eight (62.5%; 95% CI: 3.05–8.63) rhesus monkeys tested positive for hemotropic *Mycoplasma* spp. and four out of six (66.67%; 95% CI: 3.00–9.03) animals tested positive for hemotropic *Mycoplasma* spp. in T2. One rhesus monkey (#5) tested negative for hemoplasmas in T1 and tested positive in T2. All DNA samples tested negative for hemoplasmas by the 23S rRNA-PCR assay.

A total of three samples that presented strong and unique bands on electrophoresis were chosen for sequencing. From these samples, one was from T1 (#2) and two from T2 (#4 and 5). The sequence from rhesus monkey #2 obtained a size of 535 bp while sequences from rhesus monkeys #4 and #5 obtained sizes of 709 bp and 761 bp, respectively. Because of the small size sequence obtained by the sample from rhesus monkey #2 (accession nos. OK157440) was removed from further analysis. Sequences from rhesus monkeys #4 and #5 were also submitted to the GenBank^®^ database (accession nos. OK157439, OK157441). The BLASTn analysis showed that hemoplasma 16S rRNA sequences detected in NHP shared an identity ranging from 99.61% to 100% with a hemotropic *Mycoplasma* spp. (AB820288) detected in *Macaca fuscata* from Japan. The nucleotide BLASTn results were summarized on Table 1.

**Figure 1:**
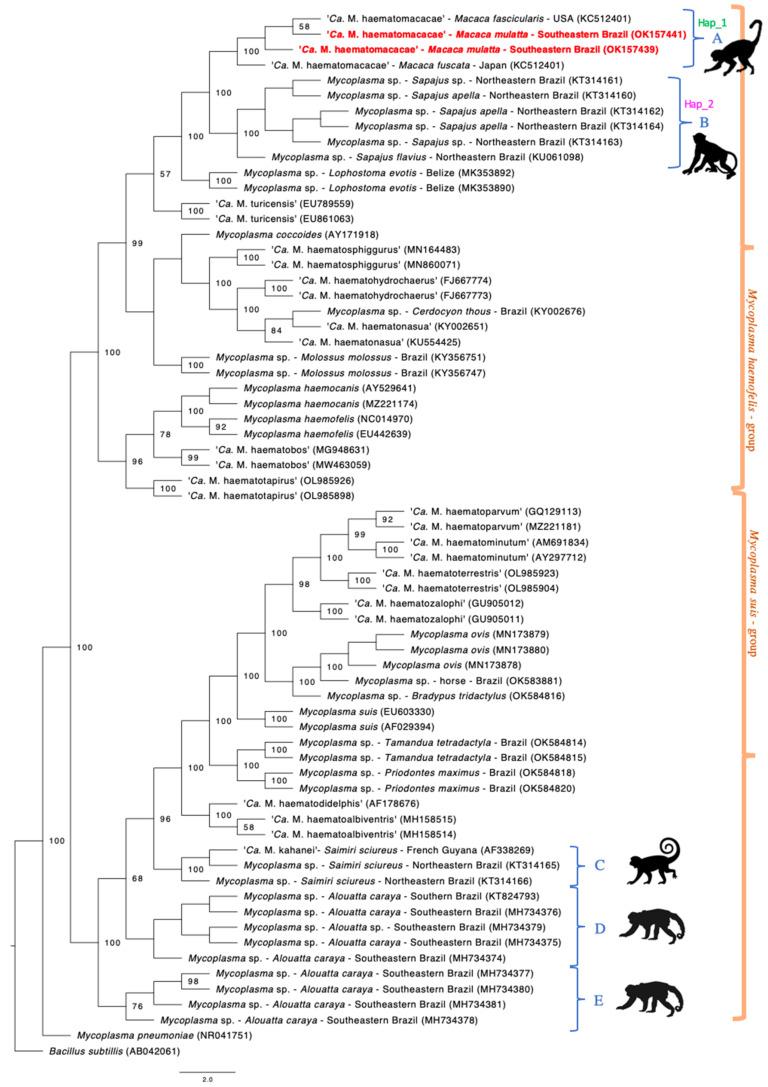
Phylogenetic tree based on Bayesian inference made with a total alignment of 750 pb and TPM3uf+I+G model. This tree demonstrates the occurrence of five different clade groups of hemoplasma genotypes occurring in free range or captive non-human primates in Brazil. Only post-probabilities values >50 are shown. Five distinct clades of hemoplasmas from NHP were formed (identified as A to E). Sequences from the present study are located in clade A and highlighted in red. The genotype groups where sequences KCS12401, AB82088, OK157439, OK157441 (Hap_1) and KT314160-KT314164, KU061098 (Hap_2) were allocated according to the genotype diversity analysis are demonstrated next to these sequences’ clades.

Phylogenetic analysis inferred by Bayesian inference and based on a 620 bp alignment (Figure 1) demonstrate the close relationship of the rhesus monkeys hemoplasma genotype with ‘*Ca*. M. haematomacacae’ genotypes detected in *M. fuscata* from Japan (GenBank^®^ accession no. AB820288) and *M. fascicularis* from the USA (KCS12401), represented by clade A. Clade B was formed involving sequences of hemotropic *Mycoplasma* sp. detected in captive *Sapajus* sp. monkeys from northeastern Brazil (KT314160, KT314161, KT314162, KT314163, KT314164, KU061098), although it clustered separately from the hemoplasma sequences detected herein. 

Another three clades were formed comprising sequences of hemotropic *Mycoplasma* spp. obtained from *Saimiri sciureus* and *Alouatta* spp. monkeys. Clade C comprised ‘*Ca*. M. kahanei’ obtained from *S. scieureus* from French Guyana (AF338269) and hemotropic *Mycoplasma* sp. from the same host species from individuals from northeastern Brazil (KT314165, KT314166). Clade D was formed with sequences of hemotropic *Mycoplasma* sp. obtained from *Alouatta* spp. monkeys from southern and southeastern Brazil (KT824793, MH734374, MH734375, MH734376, MG734379). Lastly, clade E was formed also with hemoplasma sequences of *Alouatta* spp. from southeastern Brazil (MH734377, MH734378, MH734380, MH734381).

**Figure 2:**
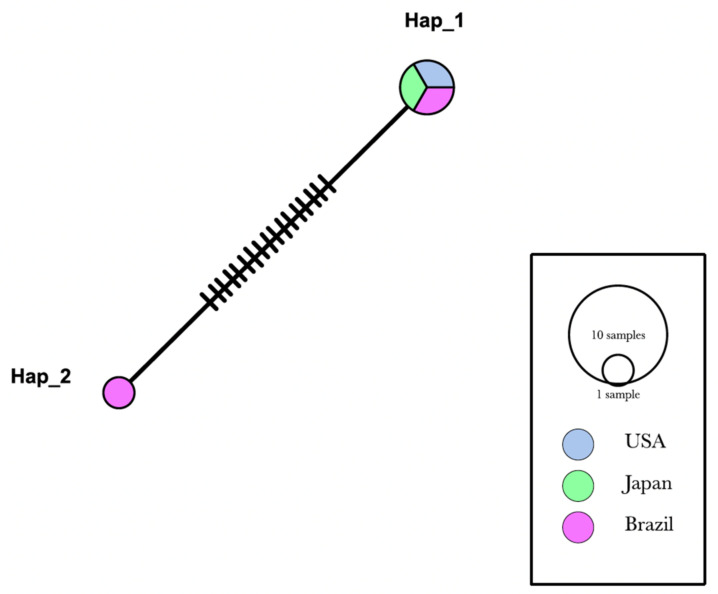
Genotype analysis made by DnaSP6 software. Inference and graphic representation were made by TCS Network method on PopART software based on a 750 bp alignment. Sequences from the present study (OK157439 and OK157441) were allocated in Hap_1.

Genotype diversity analysis (Figure 2) based on partial 16S rRNA sequences from hemotropic *Mycoplasma* sp. confirmed that the genotype that occurs in *Sapajus* spp. from Brazil are markedly different from the one occurring in *Macaca* spp. in Brazil, Japan, and the USA, with values of S = 17; k = 9.06667; Π = 0.0000056; and genotype diversity = 0.00896 (Table 2). 

Genotype Hap_1 comprised sequences of ‘*Ca*. M. haematomacacae’ isolated from *M. fascicularis* in the USA (KCS12401), ‘*Ca*. M. haematomacacae’ from *M. fuscata* from Japan (AB82088), and the sequences of hemotropic *Mycoplasma* sp. detected in rhesus monkeys #4 (OK157439) and #5 (OK157441) from Brazil described herein. All sequences obtained from *Sapajus* sp. monkeys from northeastern Brazil (KT314160, KT314161, KT314162, KT314163, KT314164, KU061098) analyzed herein were allocated in the Hap_2 genotype.

**Figure 3:**
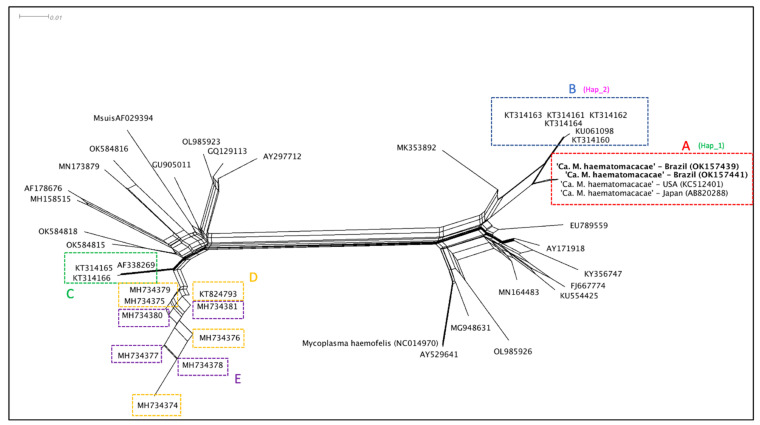
Distance analysis made by Splitstree v. 4.14.6 software applying NeighborNet method based on a 750 bp alignment. Sequences are identified based in clades formed by the phylogenetic analysis. Sequences from the present study are allocated at group A (red) together with sequences from ‘*Ca*. M. haematomacacae’ from USA and Japan. Hemotropic *Mycoplasma* sp. sequences obtained from *Sapajus* sp. monkeys from northeastern Brazil are allocated in group B (blue). Sequences of ‘*Ca.* M. kahanei’ and sequences obtained from *S. sciureus* monkeys from Brazilian Amazon are allocated in group C (green). Sequences obtained from *Alouatta* sp. from southern and southeastern Brazil are identified in yellow (those from clade C) and purple (those from clade E). The genotype groups where sequences KCS12401, AB82088, OK157439, OK157441 (Hap_1) and KT314160-KT314164, KU061098 (Hap_2) were allocated according to the genotype diversity analysis are demonstrated next to these sequences’ clade identification.

The split-network (Figure 3) analysis showed a marked difference between four genetically related groups of hemoplasmas detected in NHP from Brazil. As a part of the “*Mycoplasma suis* group”, hemotropic *Mycoplasma* sp. detected from *Alouatta* spp. monkeys from Brazil (KT824793, MH734374, MH734375, MH734376, MG734379, MH734377, MH734378, MH734379, MH734380, MH734381) and hemotropic *Mycoplasma* sp. detected from *S. sciureus* from Brazil and French Guyana (AF338269, KT314165, KT34166) formed at least two distinct groups, respectively. Meanwhile, hemotropic *Mycoplasma* sp. (KC512401, AB820288, OK157439, OK157440, OK157441) detected from *Macaca* spp. monkeys from Brazil, Japan, and the USA formed a group close to a second group where hemotropic *Mycoplasma* sp. (KT314160, KT314161, KT314162, KT314163, KT314164, KU061098) detected from *Sapajus* spp. monkeys from Brazil were allocated.

## 4. Discussion

Hemotropic *Mycoplasma* sp.-like organisms (formerly *Haemobartonella* sp.) have been previously identified on a blood smear from a splenectomised rhesus monkey coinfected with *Plasmodium cynomolgi bastianellii* [50]. Herein, we describe hemotropic *Mycoplasma* sp. infecting laboratory-kept rhesus monkeys from a research colony in Brazil through molecular methods. DNA sequencing, phylogenetic and genotype diversity analysis of partial 16S rRNA gene strongly suggest that the hemoplasma species found in the sampled rhesus monkeys was the NHP infective ‘*Ca*. M. haematomacacae’. This hemoplasma species has been firstly described in research cynomolgus monkeys (*M. fasicularis*) from the USA through analysis of partial 16S rRNA and RNase P genes [28]. Additionally, captive *M. fuscata* monkeys from Japan were found infected by a hemoplasma closely related to ‘*Ca.* M. haematomacacae’ detected in the USA [51]. 

A potentially novel hemotropic *Mycoplasma* sp. genotype (clade B) closely related to ‘*Ca.* M. haematomacacae’ has been reported infecting free-ranging and captive *Sapajus* monkeys from the northeastern region of Brazil [32,33]. Our phylogenetic and genotype diversity analysis demonstrate that hemoplasma 16S rRNA gene sequences detected in *Sapajus* monkeys from Brazil were allocated in a singular clade and genotype group, reinforcing the hypothesis that *Sapajus* sp. monkeys from Brazil are infected by a novel hemoplasma species, which should be further investigated. 

A hemotropic *Mycoplasma* genotype related to ‘*Ca*. M. kahanei’ has been reported in howler monkeys (*Alouatta* sp.) from southern and southeastern Brazil [31,34]. Based on the present Bayesian inference and distance analysis, ‘*Ca*. M. kahanei’, or even a close related genotype, may be circulating in *S. scieureus* monkeys from the Brazilian Amazon [32] (clade C). Meanwhile, two phylogenetically distinct groups of hemoplasmas appear to occur in howler monkeys from southern and southeastern Brazil (clades D and E). In contrast, splitstree analysis showed a not-so-clear separation between sequences from clade D and E. Similar results were obtained in a previous study, even with a slightly longer alignment phylogeny (800 bp) [34]. Although the Bayesian inference provided satisfactory post-probability values for separation between these clades in phylogenetic analysis, further studies using nearly complete 16S rRNA or other gene targets may be useful to fully understand the relationship between these hemoplasma genotypes.

In short, our data suggested that there is a marked difference between hemoplasma sequences from *Sapajus* sp. monkeys from northeastern Brazil and ‘*Ca*. M. haematomacacae’ from *Macaca* sp. monkeys maintained in Brazil, the USA, and Japan. Even these two groups are presented as closely related groups when compared to other hemotropic *Mycoplasma* species found in NHP (‘*Ca*. M. kahanei’ and related sequences), they apparently are not the same species.

Although hemoplasmas are reported as causative of hemolytic anemia in some host species [15], hematological abnormalities have not been associated with ‘*Ca.* M. haematomacacae’ or other related genotypes in *M. fasicularis* and *Sapajus* sp. monkeys, respectively [28,33]. A higher monocyte and lymphocyte count and a decreased count of platelets were reported in howler monkeys infected with hemoplasmas phylogenetically related to ‘*Ca*. M. kahanei’ in southeastern Brazil [34]. Hemotropic *Mycoplasma* sp. infection may lead to hematological abnormalities [25] and haptoglobin raising in apparently health hosts [52]. Thus, apparently healthy animals may not be suitable for research purposes due to latent infection effects. In the present study, one ‘*Ca*. M. haematomacacae’-infected rhesus monkey presented high values of WBC. However, it is needful to state that this individual was affected by periodontitis at the time of sampling (data not shown), which may have been responsible for the leukocytosis. Regardless, even when pathogenic infections cause clinical signs, the use of drugs to clear the pathogens may influence on animal parameters and research results [2]. Indeed, the clearance of hemoplasma blood loads is considered a defiant management, once even prolonged therapies may not result in total clearance of these pathogens [53], and combined protocols of antibiotic treatment may be needed for this purpose [54], reinforcing the need to use only pathogen-free animals for research purposes. 

Sampling the same animals in two different times allowed us to observe that rhesus monkeys apparently maintained the hemoplasma infection through at least 10 months. Chronical infections of hemotropic *Mycoplasma* spp. are already reported in domestic [54,55] and wild animals [56] and usually occur in seemingly healthy and non-splenectomised hosts [15]. In addition, rhesus #5 presented negative results in T1 and became positive in T2, suggesting that this particular animal had been infected between samplings or even that in T1 the bacterial load may be below the threshold detection from the conventional PCR assay used herein. 

Transmission routes for hemoplasmas are still not elucidated. Although there is no robust evidence to support that hemotropic mycoplasmas are vector-borne pathogens, recent studies have reported detections of hemoplasma DNA in arthropods such as salivary glands of *Amblyomma dubitatum* ticks [57] and *Polyplax spinulosa* lices [58]. Sampled animals from the present study received a monthly application of ivermectin, and ectoparasite infestations were not reported. Previous studies on *M. haemomuris* have showed that hemoplasma latent infections may occur in laboratory animals with an absence of ectoparasites [16], and transplacental transmission is also concerned [26]. In studies conducted in rodents, direct contact between hosts was associated with the transmission of hemoplasmas [59]. The transmission of hemotropic *Mycoplasma* sp. between monkeys in research colonies may be associated with contaminated needles or nasogastric tubes [60]. Although the hypothesis that the transmission of ‘*Ca*. M. haematomacacae’ herein may be associated with experimentational manipulation or direct transmission through aggressive interaction between NHP, it is not possible to strongly link it with the results found. In all cases, our data highlight the importance of ensuring biosecurity proceedings and preventive screenings aiming to avoid hemoplasma transmission through research monkey colonies. For this purpose, molecular techniques are suggested due its increased sensitivity [15].

A previous study has showed that *M. coccoides* may alter mice hosts resistance to Newcastle disease virus, Chikungunya virus [17,22], and malaria [18,61,62], with co-infections accounting for the variation in virulence of *Trypanosoma brucei* infection in these hosts [19]. On this pattern, considering that rhesus monkeys evaluated herein are used for research purposes, it is important to investigate whether ‘*Ca*. M. haematomacacae’ may represent an important bias on future studies. 

Limitations from the present study should be considered. Studies based on the entire 16S rRNA gene and the intergenic spacer region between 16S and 23S rRNA genes showed that a monkey colony was entirely infected by the same strain of ‘*Ca*. M. haematomacacae’ in Japan [51]. Even that a small sample size was analyzed herein due convenience, the present study demonstrates that 62.5% of rhesus monkeys maintained in a research colony have tested PCR-positive for hemoplasmas. Unfortunately, it was not possible to sequence more samples or amplify other gene targets to evaluate the genetic diversity between the detected hemoplasmas. Additionally, short-read sequencing platforms targeting partial regions of 16S rRNA may not achieve full taxonomic resolutions that are more consistently obtained by full-length sequencing [63]. Considering that the blood sampling on rhesus monkeys sampled herein have initially aimed health assessment purposes, we highlight the importance on conducting molecular diagnosis for hemoplasma detection in animals used for research purposes.

## 5. Conclusions

‘*Candidatus* M. haematomacacae’ was found in laboratory-kept rhesus monkeys from a research colony in Brazil. Although no hematological abnormalities may be associated with ‘*Ca.* M. haematomacacae’ in assessed animals, future studies are needed to elucidate the influence of this hemoplasma species as a bias on research studies. Finally, the assessment of the genotype diversity of hemoplasma species in NHP from Brazil suggested that ‘*Ca*. M. haematomacacae’ and *Sapajus*-related hemotropic *Mycoplasma* sp. found in NHP from northeastern Brazil are genetically close but divergent species based on partial 16S rRNA analysis.

## Figures and Tables

**Table 1:** Sampled animals from the present study, results for *Mycoplasma* spp. 16S rRNA amplification, higher similarities found in GenBank, query cover and E-value results obtained from BLASTn analysis; NS = ‘No sample’—animal died before the second sampling; bp = base pair.

Animal	T1	T2	Sequence Size (bp)	BLASTn Identity	Query Cover (%)	E Value
1	Negative	Negative	-	-	-	-
2	**Positive**	NS	535	99.81%—’*Ca.* M. haematomacacae’ from Japan (AB820288)	100	0.0
3	**Positive**	**Positive**	-	-	-	-
4	**Positive**	**Positive**	709	100%—‘*Ca.* M. haematomacacae’ from Japan (AB820288)	100	0.0
5	Negative	**Positive**	761	99.61%—‘*Ca.* M. haematomacacae’ from Japan (AB820288)	100	0.0
6	**Positive**	NS	-	-	-	-
7	**Positive**	**Positive**	-	-	-	-
8	Negative	Negative	-	-	-	-

**Table 2:** Genotype analysis values obtained by DnaSP6 software.

Number of Sequences (N)	Number of Genotypes (*h*)	Number of Variable Sites (S)	Average Number of Nucleotide Differences (k)	Π (Mean (SD))	Genotype Diversity (Mean (SD))	G + C Content (%)
10	2	17	9.06667	0.0000056 [0.00236]	0.00896 [0.095]	46.4

## Data Availability

Nucleotide sequences obtained in the present study can be found at Genbank data base (https://www.ncbi.nlm.nih.gov/genbank/, accessed on 7 July 2022) under accession numbers: OK157439, OK157440, and OK157441.
